# IDENTIFYING PHENOTYPES OF OLDER ADULTS PLANNING FOR ALZHEIMER’S DISEASE SUPPORT

**DOI:** 10.1093/geroni/igae098.2275

**Published:** 2024-12-31

**Authors:** Amber Miller-Winder, Vanessa Ramirez-Zohfeld, Raven Relerford, Allison Schierer, Charlie Olvera, Alaine Murawski, Lee Lindquist

**Affiliations:** Northwestern University, Evanston, Illinois, United States; Northwestern University, Evanston, Illinois, United States; Northwestern Medicine, Chicago, Illinois, United States; Northwestern University Feinberg School of Medicine, Chicago, Illinois, United States; Northwestern University Feinberg School of Medicine, Division of Geriatrics, Evanston, Illinois, United States; Northwestern University, Evanston, Illinois, United States; Northwestern University, Evanston, Illinois, United States

## Abstract

Older adults (OAs) with Alzheimer’s Disease (AD) often need additional support (e.g. in-home caregivers) but are unprepared. We modeled the types of plans OAs make surrounding long-term care (LTC)/ home support in the event of AD to describe different planning phenotypes. The Plan Your Lifespan (PYL) study examines OAs’ decision-making process about LTC/home support in the event of AD. Subjects are surveyed every 6 months about cognitive, social, functional, environmental factors; including living/support preferences. We employed mixed analytic methods (qualitative coding and GLMM). 293 subjects enrolled (mean age 73.5 yrs,72.7% female, 40.4% URM). Almost half (47.4%, n=139) experienced cognition decline with 10.3% identifying worsening memory loss. We identified four planning phenotypes: Deniers overlook the possibility of developing AD. Subjects with no measured cognitive impairment were 35% less likely to plan; Do-gooders plan to avoid burdening family and often complete an Advanced Directive (OR 1.75 [p < 0.05, 1.20-2.54]) or Living Will (OR 1.56 [p < 0.05, 1.08-2.25]; Defeaters do not plan due to external limitations; a below average Consumer Health Activation score (OR 0.65 [p < 0.05, 0.43-0.98]) signaled 35% less likely to plan; and Dumpers leave planning to their family; displaying sufficient social support (OR 2.4 [p < 0.05, 1.39-4.15]) and limited health literacy (OR 1.99, [p < 0.05, 1.24-3.2]). Helping OAs consider their support needs through identification of different phenotypes may allow providers to intervene early. Phenotypes depend on health literacy, social support, self-efficacy, and finances. Further research will examine how planning phenotypes change over time and how plans are implemented to best care for OAs with AD.

